# Variation in the ontogenetic allometry of horn length in bovids along a body mass continuum

**DOI:** 10.1002/ece3.6181

**Published:** 2020-03-27

**Authors:** Morgane Tidière, Jean‐Michel Gaillard, Mathieu Garel, Jean‐François Lemaître, Carole Toïgo, Christophe Pélabon

**Affiliations:** ^1^ Department of Biology Centre for Biodiversity Dynamics NTNU Norwegian University of Science and Technology Trondheim Norway; ^2^ Laboratoire de Biométrie et Biologie Evolutive - UMR5558 - CNRS Université Claude Bernard Lyon 1 Université de Lyon Villeurbanne France; ^3^ Office Français pour la Biodiversité Gières France

**Keywords:** comparative analysis, development, ornament, sexual selection, ungulates, weapons

## Abstract

Allometric relationships describe the proportional covariation between morphological, physiological, or life‐history traits and the size of the organisms. Evolutionary allometries estimated among species are expected to result from species differences in ontogenetic allometry, but it remains uncertain whether ontogenetic allometric parameters and particularly the ontogenetic slope can evolve. In bovids, the nonlinear evolutionary allometry between horn length and body mass in males suggests systematic changes in ontogenetic allometry with increasing species body mass. To test this hypothesis, we estimated ontogenetic allometry between horn length and body mass in males and females of 19 bovid species ranging from ca. 5 to 700 kg. Ontogenetic allometry changed systematically with species body mass from steep ontogenetic allometries over a short period of horn growth in small species to shallow allometry with the growth period of horns matching the period of body mass increase in the largest species. Intermediate species displayed steep allometry over long period of horn growth. Females tended to display shallower ontogenetic allometry with longer horn growth compared to males, but these differences were weak and highly variable. These findings show that ontogenetic allometric slope evolved across species possibly as a response to size‐related changes in the selection pressures acting on horn length and body mass.

## Graphical abstract

A general observation about living organisms is the tendency for variation in morphological, physiological, and life‐history traits to be highly correlated with variation in organism size, a phenomenon referred to as allometry. It remains largely unknown, however, whether these patterns of covariation at the among‐species level (evolutionary allometry) result from selection or developmental constraints. We studied variation in ontogenetic allometry between horn size and body mass in males and females of several bovid species. Ontogenetic allometry changed systematically with an increase in species mass, from steep allometries with short period of horn growth in small species to shallow allometry with extended horn growth in large species. These findings suggest that ontogenetic allometry evolved across species as a response to size‐related changes in the selection pressures acting on horn length and body mass, respectively. They further support the adaptive origin of evolutionary allometry between horn size and body mass in Bovids.

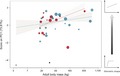

## 1 INTRODUCTION

Allometric relationships describe patterns of proportional covariation between morphological, physiological, or life‐history traits and the size of the organisms among populations or species (evolutionary allometry), or within population, among individuals measured at similar (static allometry), or different (ontogenetic allometry) age or developmental stages. When expressed on a log–log scale, allometric relationships are often described by a linear regression: log(*y*) = *a* + *b* log(*x*) where *y* is the trait size; *x* the body size; and *a* and *b* the allometric intercept and slope, respectively (Huxley, 1932). Because population and species mean trait size and body size used to estimate evolutionary allometry result from the proportional growth of both traits, patterns of evolutionary allometry emerge from variation in ontogenetic allometry (Cheverud, 1982; Gould, 1966; Klingenberg & Zimmermann, 1992). More specifically, evolutionary allometric slopes and intercepts are determined by patterns of variation and covariation between ontogenetic allometric parameters (slope and intercept) and body size (Pélabon et al., 2013).

**Figure 1:**
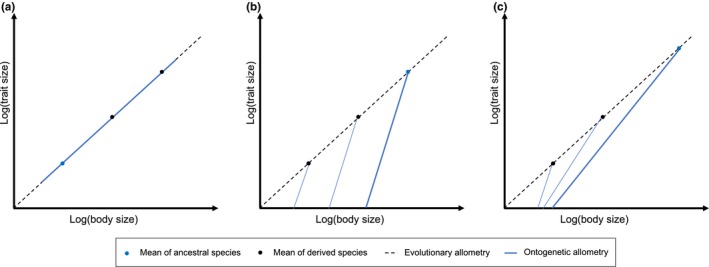
Different evolutionary scenario linking changes in ontogenetic allometry to patterns of evolutionary allometry. (a) Ontogenetic scaling: extension of the ancestral ontogenetic trajectory among species (no variation in ontogenetic allometric intercept and slope). (b) Heterochrony: acceleration or retardation of the development generating changes in the ontogenetic intercept (here acceleration of the development, i.e., later developmental stages appear at younger age). (c) No‐constraint scenario with changes in ontogenetic slope and intercept

It remains largely unknown, however, how evolvable are parameters of ontogenetic allometry, and particularly how evolvable are ontogenetic allometric slopes. On the one hand, the weak evolutionary changes often observed in static allometric slopes (Voje, Hansen, Egset, Bolstad, & Pélabon, 2014) as well as the difficulties to change these slopes via artificial selection (Bolstad et al., 2015; Egset et al., 2012) suggest that allometric slopes, whatever the taxa considered, have low evolutionary potential and may represent evolutionary constraints (Houle, Jones, Fortune, & Sztepanacz, 2019; Maynard‐Smith et al., 1985; Pélabon et al., 2014; Voje et al., 2014). On the other hand, among‐species variation in ontogenetic allometric slopes reported by some studies suggests that these slopes may be evolvable (Klingenberg & Froese, 1991; Urošević, Ljubisavljević, & Ivanović, 2013; Weston, 2003). Yet, despite the plethora of studies on ontogenetic, static, and evolutionary allometry, variation in ontogenetic allometric parameters and the consequence of this variation on static and evolutionary allometry is poorly known. Consequently, it remains uncertain whether evolutionary allometry mostly results from ontogenetic scaling, that is, the extension of the ancestral allometric trajectory among species with no variation in slope or intercept (Figure 1a; Corner & Shea, 1995; Shea, 1983; Weston, 2003), heterochrony, that is, the acceleration or retardation of the development that generates changes in the ontogenetic intercept (Figure 1b, Gould, 1971, 1977) or changes in both ontogenetic slope and intercept (Figure 1c, Gould, 1966). Distinguishing between these different scenarios that represent different levels of evolutionary constraints is difficult, however, because the invariance of allometric parameters may also result from the consistency of the selection pressures among populations or species.

In a recent study, Tidière, Lemaître, Pélabon, Gimenez, and Gaillard (2017) showed that the evolutionary allometry between horn length and body mass in male bovids is nonlinear, the allometric slope decreasing with an increase in body mass across species. This pattern was interpreted as evidence for a shift in the target of sexual selection in males, from horn length to body mass, when the species body mass increases. If this hypothesis is true, the change in the target of sexual selection should generate changes in the patterns of ontogenetic allometry between horn length and body mass from small to large species, thus offering an excellent opportunity to test how evolvable are ontogenetic allometric parameters. Similarly, systematic changes in ontogenetic allometry are also expected between sexes due to the different roles supposedly played by horns in male and female bovids (Darwin, 1871; Roberts, 1996).

To test these hypotheses, we collected data on horn length and body mass growth in males and females of bovid species. We obtained data from 22 populations of bovids encompassing 19 extant species ranging from 4.6 (*Madoqua kirkii*) to 717 kg (*Syncerus caffer*; Table 1). For each species and population, we estimated sex‐specific ontogenetic allometries and tested whether these allometries changed in response to variation in body mass, and whether such a change differed between sexes.

**Table 1:** Ontogenetic allometry parameters and key life‐history traits for males and females of 19 bovid species

Species	Subspecies, population	Original study	Sex	Alpha	*SE*	*β*_1_	*SE*	*β*_2_	*SE*	Adj.‐*R* ^2^	*T*_BM_ (kg)	95% CI	Tp (%)	*N* ind	*N* dots	BM_max_ (kg)	BM_0_ (kg)	Range BM (%)
*Aepyceros melampus*	Nyala Game Ranch	Anderson (1982)	m	−1.83	0.35	1.54	0.10	0.02	0.45	0.996	**40.33**	39.53; 41.14	0.98	50	4	44.2	5.6	49.1
*Bison bonasus*	Bialowezia	Krasińska and Krasiński (2002)	m	−1.27	0.26	0.86	0.05	0.02	0.08	0.989	**350.72**	330.30; 399.41	0.91	>100	6	634	23.4	99.6
			f	0.58	0.18	0.51	0.03	15.85	7.90	0.988	422.00	374.28; 424.0	1.00	>100	6	424	23.4	99.4
*Capra ibex*	Belledonne	ONCFS	m	−1.18	0.13	1.26	0.03	0.28	0.54	0.924	86.83	80.16; 109.00	0.95	136	136	109	2.9	100
			f	−0.87	0.13	1.08	0.04	0.31	0.19	0.872	**42.35**	40.29; 46.34	0.93	166	166	57	2.9	99.9
*Capra pyrenaica*	Sierra de Cazorla	Fandos and Vigal (1988)	m	−0.65	0.26	1.18	0.07	36.71	28.23	0.963	56.04	52.77; 56.30	1.00	>35	13	56.3	11.3	100
			f	−1.42	0.47	1.25	0.15	0.40	0.69	0.872	**29.11**	23.36; 36.27	0.94	>25	15	36.5	10.7	100
	Sierra de Gredos	Fandos and Vigal (1988)	m	−0.81	0.51	1.21	0.14	8.84	97.10	0.884	71.02	57.00; 71.20	1.00	>35	12	71.2	12.7	100
			f	−1.40	0.49	1.27	0.15	−2.65	3.17	0.865	37.08	33.21; 39.50	0.98	>25	13	39.5	8.6	100
*Damaliscus lunatus*	*D.l. lunatus*, North. Botswana	Child, Robbel, and Hepburn (1972)	m	−4.50	0.23	1.73	0.06	0.22	0.26	0.989	**102.21**	97.22; 108.53	0.94	>50	17	140.8	14.4	97.1
			f	−13.33	0.77	4.07	0.20	0.30	0.16	0.991	**59.68**	56.77; 63.37	0.84	>60	13	126.7	14.3	86.4
*Gazella arabica*	*G.a. erlangeri*, KKWRC	Wronski et al. (2010)	m	−0.98	0.47	1.68	0.22	0.02	0.32	0.837	**11.36**	10.18; 12.94	0.84	20	20	18	2.6	84.4
			f	−0.87	1.20	1.10	0.55	0.17	1.45	0.597	14.34	6.01; 19.00	0.90	7	7	19	2.6	79.3
	KKWRC	Wronski et al. (2010)	m	−2.93	0.88	2.09	0.32	0.05	0.48	0.621	**18.62**	16.35; 21.20	0.89	37	37	27	2.6	69.7
			f	−0.37	0.43	1.01	0.16	−0.15	0.98	0.321	18.92	16.44; 22.00	0.95	91	91	22	2.6	87.6
*Gazella dorcas*	KKWRC	Wronski et al. (2010)	m	−2.23	0.91	1.91	0.34	−0.08	12.45	0.850	19.65	16.74; 20.00	0.99	8	8	20	1.7	59.9
			f	−6.09	4.22	3.51	1.72	0.42	1.51	0.297	**13.12**	12.23; 16.84	0.91	16	16	17	1.7	45.6
*Gazella subgutturosa*	*G.s. marica*, KKWRC	Wronski et al. (2010)	m	−5.07	0.25	2.98	0.10	0.50	0.23	0.898	**16.61**	16.12; 17.64	0.84	150	150	28	2.6	90.6
			f	−4.60	0.41	2.84	0.16	0.18	0.26	0.639	**14.73**	14.15; 15.49	0.85	229	229	24	2.6	88.9
*Gazella thomsoni*	Northern Tanzania	Robinette and Archer (1971)	m	−8.39	0.16	3.75	0.05	0.38	0.86	0.999	**23.43**	23.20; 23.67	0.98	101	8	28	2.6	79.9
			f	−9.27	0.66	3.95	0.23	0.26	1.50	0.984	**19.55**	18.78; 20.15	0.98	159	7	21.5	2.6	73.5
*Hemitragus jemlahicus*	New Zealand	Parkes and Tustin (1989)	m	−2.25	0.01	0.99	0.00	1.09	0.44	1.000	73.41	56.20; 74.00	1.00	157	6	74	2	69.8
*Madoqua kirkii*	*M.k. thomasi*	Hutchison (1970)	m	−9.32	1.73	11.11	2.09	1.22	1.95	0.898	**2.57**	2.37; 2.84	0.79	2^a^	7	4.6	0.63	29.8
*Oreamnos americanus*	Caw Ridge Alberta	Festa‐Bianchet and Côté (2008)	m	−0.86	0.29	0.97	0.08	0.08	0.06	0.980	**59.44**	55.98; 63.75	0.88	>100	8	105.6	3.2	68.7
			f	−0.78	0.33	0.94	0.09	0.16	0.31	0.950	**64.01**	58.50; 70.04	0.97	>40	12	73.7	3.2	58.5
*Ovis aries*	St.Kilda	Robinson, Pilkington, Clutton‐Brock, Pemberton, and Kruuk (2006)	m	−0.84	0.17	1.31	0.05	0.20	0.46	0.990	**36.67**	35.23; 39.33	0.97	>100	9	40.8	2.4	68.1
			f	0.08	0.22	0.86	0.07	5.47	5.96	0.916	22.90	21.78; 23.10	1.00	>100	15	23.1	2.4	55.1
*Ovis gmelini*	*O.g. musimon* × *Ovis* sp., Caroux‐Espinouse	ONCFS	m	−3.41	0.13	2.13	0.04	0.33	0.19	0.877	**32.75**	31.47; 34.43	0.88	442	442	53	2.5	100
*Redunca fulvorufula*	Cape province	Norton and Fairall (1991)	m	−23.80	3.39	8.04	1.08	0.38	1.03	0.921	**26.50**	24.22; 27.85	0.92	115	12	35.2	3	64.1
*Rupicapra rupicapra*	Western Italian Alps	Bassano, Perrone, and Hardenberg (2003)	m	1.11	0.08	0.62	0.02	2.55	2.77	0.989	27.63	25.76; 27.63	1.00	337	9	27.8	2.4	51.6
			f	−0.48	0.48	1.15	0.16	−0.10	0.58	0.871	**20.68**	19.28; 22.18	0.97	176	9	22.8	2.4	39.7
	Abruzzo National Park	Locati and Lovari (1991)	f	−1.67	0.98	1.43	0.32	0.46	0.59	0.878	24.90	22.76; 29.50	0.95	7	7	29.5	2.4	38.7
*Sylvicapra grimmia*	Zimbabwe	Wilson, Schmidt, and Hanks (1984)	m	−7.08	0.50	4.56	0.22	0.87	0.21	0.980	**11.74**	11.39; 13.11	0.85	5	22	18.1	1.6	69.2
*Syncerus caffer*	Kruger National Park	Geist and Walther (1974)	m	1.67	0.42	0.44	0.07	10.30	5.13	0.975	714.8	693.67; 717.60	1.00	37	5	717.6	44	31.1
*Tragelaphus strepsiceros*	Eastern Cape	Prinsloo and Jackson (2017)	m	−6.83	0.74	2.25	0.15	0.16	0.28	0.957	**184.01**	168.17; 193.35	0.93	460	16	280	13	100

Note

For each sex, population, and species, parameters (±*SE*) were estimated from a segmented regression model. These parameters are as follows: the intercept (*α*, in log(cm)), the slope before the threshold mass (*β*_1_), the slope after the threshold mass (*β*_2_), the absolute threshold mass (*T*_BM_ in %, and its 95% confidence interval)). We also report the adjusted *R* ^2^ of the model, the total number of data points per age class used to measure the ontogenetic allometry (*N* dots), and the number of individual measures available (*N* ind). Thresholds reported in bold are statistically different from the maximum body mass in the dataset. The sex‐ and population‐specific maximum adult body mass (BM_max_) have been obtained from the original study except in *M. kirkii* (collected from Bro‐Jørgensen, 2007). From BM_max_ and *T*_BM_, we calculated the proportional threshold mass (Tp) as *T*_BM_/BM_max_. Birth mass (BM_0_) has been collected in the original study when available and from AnAge (Tacutu et al., 2012) in other cases (except for *M. kirkii*, Hutchison, 1970) and *O.g. musimon* (M.G, unpubl. data)). From BM_max_ and BM_0_, we estimated the proportion of the total body mass range that was covered by the data (Range BM).

^a^ 2 individuals have been measured repeatedly all along their growth.

## 2 MATERIAL AND METHODS

### 2.1 Data collection

We conducted a literature survey using the keywords “*horn*” or “*weapon*” in combination with the genus names of the 137 extant bovid species in the core collection of ISI Web of Knowledge and Google Scholar. We retained only studies that presented direct measurements of horn length and body mass. The literature search yielded 16 studies including data for 17 bovid species from which we could estimate the ontogenetic allometry for males and/or females. These data corresponded to either means per age class reported in tables or individual data on horn length or body mass presented in tables or graphs. For measurements given per age class, we only considered studies with a minimum of four age classes. We extracted data from graphs using WebPlotDigitizer version 4.1. For one study including three species (four populations) of the genus *Gazella* (Wronski, Sandouka, Plath, & Cunningham, 2010), we obtained the raw data directly from the first author. For seven out of 16 studies, graphs displayed horn length or body mass as a function of age without linking individual data points for the two variables. This prevented us to estimate directly ontogenetic allometry from the data. We thus calculated the average horn length and body mass per age class and estimated ontogenetic allometry from these average values. We did not include age classes for which horns were not yet present.

We also included in our analysis unpublished data provided by the French Hunting and Wildlife Agency (ONCFS) for the Alpine ibex (*Capra ibex*) and the Mediterranean mouflon (*Ovis gmelini musimon* × *Ovis* sp.)*.* These data correspond to individual data for horn length and body mass obtained from long‐term population monitoring (Garel et al., 2007; Toïgo, Gaillard, & Loison, 2013).

In total, we collected data on horn length and body mass for 19 bovid species (19 species for males with two species including two different populations, and 11 species for females with three species including two different populations). This yielded a total of 35 ontogenetic allometries (Table 1). Data come from wild or semiwild (unfed) populations except for males of *M. kirkii* (Table 1) for which data were obtained from two calves captured in the wild and raised in captivity hand‐fed. Horn length is generally measured from the tip to the base of the horn using a flexible ruler placed along the external curvature of the horn (see Table S1). Although this method may slightly differ among studies, these differences are not expected to affect the results because allometry measures the proportional change in horn size for a proportional change in body mass. Therefore, as long as the measurement method captures the increase in horn length, it provides an estimate of the allometric relationship between horn size and body mass comparable across species.

### 2.2 Data analysis

#### 2.2.1 Estimating ontogenetic allometry

Ontogenetic allometry is often nonlinear (Deacon, 1990; Pélabon et al., 2013) and can be described by various models such as quadratic or segmented regressions (i.e., threshold models) or standard asymptotic growth models (e.g., monomolecular, Gompertz, logistic, or von Bertalanffy, France, Dijkstra, & Dhanoa, 1996). Segmented regressions are seldom used in allometric studies (but see Huxley, 1932; Knell, 2008; Lemaître, Vanpe, Plard, & Gaillard, 2014; McCullough, Ledger, O'Brien, & Emlen, 2015). Segmented regressions are described by four parameters: the intercept (*α*), the slope before the threshold (*β*_1_), the position of the threshold on the *x*‐axis at which the slope shifts (*T*_BM_, in natural log), and the slope after the threshold (*β*_2_). The threshold represents the point during ontogeny at which the ratio between the proportional growth of the organ and the body size (i.e., the allometric slope) changes. This occurs, for example, when the growth of the organ stops or slows down, while the increase in body size is sustained or accelerates (e.g., allometry in brain size in mammals before and after birth, Deacon, 1990), or if the growth of the organ accelerates (e.g., the tail sword in male guppies after sexual maturation, Egset et al., 2012).

Comparing nonlinear allometric relationships using quadratic regressions among species is complicated by the fact that the slope changes when body mass increases, thus preventing comparison of the allometric slope among species with different body mass. With segmented regressions, allometric slopes estimated before and after the threshold correspond to homologous growth periods that can be compared between sexes, populations, or species with different body mass. This method also allows comparing linear with nonlinear ontogenetic allometry because the former corresponds to a model where the threshold occurs at the maximum body mass (see Appendix S1 for further details). We thus described ontogenetic allometry of horn length in bovids using segmented regressions.

We fitted segmented linear regressions on a log–log scale for each sex in each population (see Figure S1 for graphical representations of the models fitted on the raw data for each sex of each population). The threshold and its 95% confidence interval were estimated by maximum likelihood (Ulm & Cox, 1989, see Appendix S2 for the R script). Ontogenetic allometries were thus characterized by four parameters *α*, *β*_1_, *T*_BM_, and *β*_2_. However, *β*_2_ was not considered further in the analyses because it was not statistically different from zero in most cases (see Results). To compare the value of the threshold between sexes and among populations and species, we expressed it as a proportion of the final body mass (Tp = *T*_BM_/BM_max_ where BM_max_ is the maximum body mass). For *M. kirkii*, the maximum body mass reported in the study was lower than the adult body mass reported in the literature for the species. Therefore, we used the latter to calculate Tp (Table 1).

The use of log‐transformed data in allometry studies has been criticized (Packard, 2018), but this method is totally justified by the multiplicative nature of the residual variation in ontogenetic allometry (Kerkhoff & Enquist, 2009; Pélabon, Tidière, Lemaître, & Gaillard, 2018). Furthermore, only linear regressions on a log–log scale allow comparing allometric slopes of species with different body mass (Pélabon et al., 2018, see also Appendix S1). Because body mass is a function of the volume of the organism and horn length is a linear measurement, isometry between horn length and body mass corresponds to an allometric slope of 1/3 on a log‐log scale. 

The precision of the parameter estimates for ontogenetic allometry depends on the range of body mass over which measurements are performed. Shorter is the range, lower is the precision, particularly for the threshold. Differences in the range of body mass used to estimate ontogenetic allometry may generate heterogeneity among species or bias the analysis if the range is correlated with the size of the species. We thus estimated the range of body mass for each data set by collecting sex‐ and population‐specific adult and neonate body mass either from the same studies as the ones providing the data on ontogenetic allometry or from other sources including the database AnAge (Tacutu et al., 2012, Table 1). For all species, except *M. kirkii* (see above), the maximum body mass reported in the original studies corresponded to the adult body mass.

#### 2.2.2 Analyzing variation in ontogenetic allometry

We first assessed patterns of variation in ontogenetic allometries by estimating variation and covariation in *α*, *β*_1_, and Tp. Because these three parameters were strongly correlated (see Results), we ran a normed principal component analysis (PCA, package “*ade4*,” Dray & Dufour, 2007) and used the scores of the first principal component (PC1) as a “shape index”: a measure of variation in ontogenetic allometry between horn length and body mass across bovids. To test the effects of body mass and sex on ontogenetic allometry, we ran mixed effect linear model using the function “*lmer*” (package “lme4,” Bates et al., 2015), where PC1 scores were the response variable, sex‐ and sex‐specific mean adult body mass and their interaction were predictor variables, and species were fitted as random effect to account for the fact that species were sometimes represented by several populations or/and by males and females. We also included the range of body mass covered by each data set (in percent) as predictor variable to correct for the possible bias in the parameter estimates of the ontogenetic allometry generated by a small range of data. We used the inverse of the variance in the ontogenetic slope *β*_1_ (i.e., squared *SE* of *β*_1_) as a weighting factor to account for variation in the precision of the estimates (Burnham, 1987). We were not able to perform an analysis controlling for phylogenetic relationship among species due to the small number of species considered (i.e., 19 species). Indeed, analyzing less than 20–25 species prevents a robust estimation of phylogenetic inertia (see e.g., Sæther et al., 2013 for a similar argumentation). Finally to assess whether the observed variation in ontogenetic slopes exceeded that expected from the sampling variance, we computed the standard deviation of the ontogenetic slopes, controlled for uncertainty, as $\sigma_{b}=\sqrt{Var\left(b\right)-\bar{SE_{b}^{2}}}$ , where *Var(b)* is the variance of the slopes among sexes, populations, and species, and $\bar{SE_{b}^{2}}$ is the average squared standard error of the slope estimates. If $\sigma_{b}$ is positive and defined, it means that the variation in ontogenetic slope is stronger than the variation only due to sampling error.

All the analyses have been performed with R version 2.14.0 (R Core Team, 2018), and we provide here parameter estimates ± *SE* or 95% confidence intervals.

## 3 RESULTS

**Figure 2:**
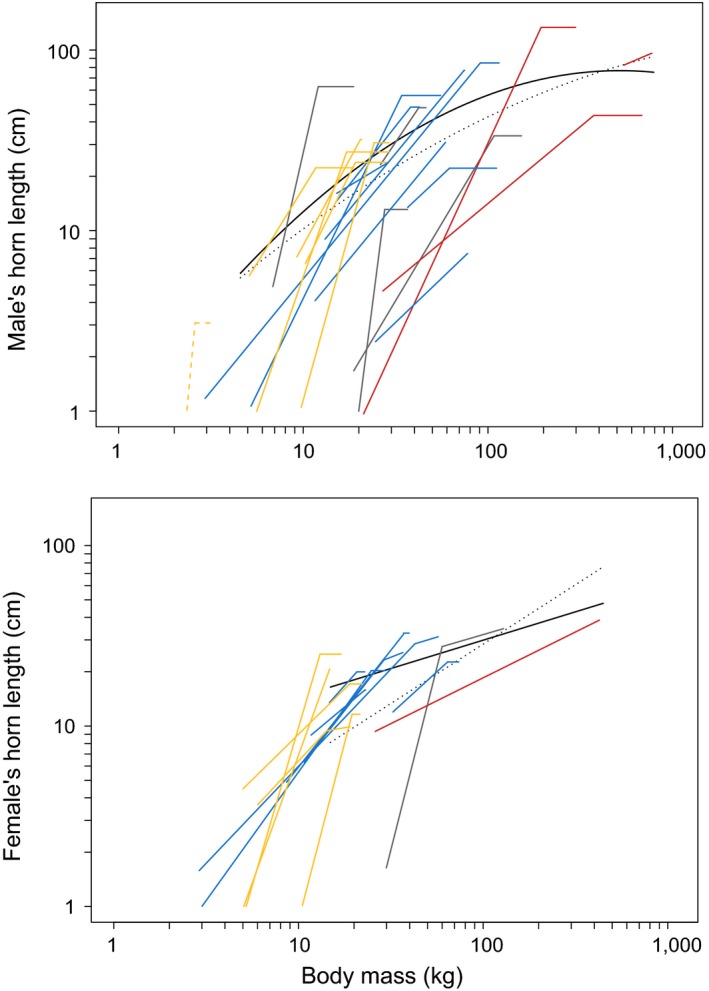
Evolutionary (black solid line, males: log(horn length) = −0.65 + 1.46 * log(body mass) − 0.12 * log(body mass)^2^, females: log(horn length) = 0.32 + 0.66 * log(body mass), Table S3) and ontogenetic allometries (on log‐log scale) for males and females of 19 bovid species (35 different populations). Antilopinae are represented in yellow, Bovinae in red, Caprinae in blue, and other species in gray. For comparison, the evolutionary allometry obtained in Tidière et al. (2017) is reported (dotted line, Table S3)

Ontogenetic allometries varied among species as a result of variation in the intercept *α*, the allometric slope before the threshold *β*_1_, and the absolute threshold *T*_BM_ (Figure 2; Figure S2). The allometric slope was generally steeper than isometry (median = 1.31, to be compared to 0.33) and ranged from 0.44 (*SE* = ±0.07) for *S. caffer* males to 11.11 (*SE* = ±2.09) for *M. kirkii* males (Figure S2). The standard deviation in ontogenetic slope corrected for sampling variance is positive and equals 2.10, confirming that the variation in ontogenetic slope observed among sexes, populations, and species is stronger than the variation expected by sampling variance alone.

Allometric slopes after the threshold (*β*_2_) were rarely statistically different from zero (Table 1). Therefore, the threshold indicates the body mass at which horn length stops increasing. This threshold ranged from 79% of the maximum body mass for *M. kirkii* males to 100% for *Bison bonasus* females (median = 94.53%, Figure S2) and was lower than the maximal body mass (i.e., maximum body mass not included in the 95% CI of *T*_BM_) in 67% of the cases for males and 57% of the cases for females. The intercept also varied among species, from −23.80 (*SE* = ±3.39) for *Redunca fulvorufula* males to 1.67 (*SE* = ±0.42) for *S. caffer *males (Figure S2). For two species (*M. kirkii* and *R. fulvorufula*, Figure S2), the allometric parameters were outliers. To assess the robustness of our results, we performed the subsequent analyses with and without these two extreme points but results remained qualitatively unchanged.

As expected, allometric slopes and intercepts were negatively correlated (Table S2). We also observed a negative correlation between the allometric slope and the proportional threshold Tp, whereas the intercept and the threshold were positively correlated (Table S2). The negative correlation between the ontogenetic slope *β*_1_ and the threshold Tp revealed a gradient of ontogenetic allometry going from species exhibiting a rapid horn growth relative to body mass with an early cessation of the horn growth (steep allometry with low threshold, e.g., males of goitered gazelle, *Gazella subgutturosa*) to species exhibiting a slower (relative to body mass) but prolonged horn growth (i.e., shallow allometry and late threshold, e.g., *B. bonasus* females).

**Figure 3:**
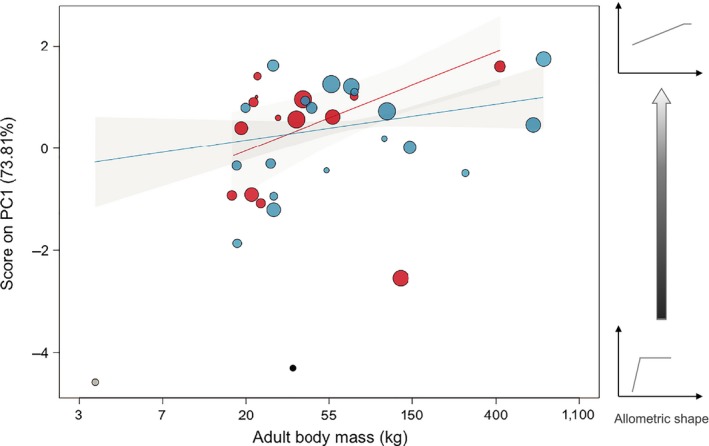
Positive relationship between the PC1 of the PCA (including intercept *α*, allometric slope *β*_1_, and threshold Tp), maximum body mass (on log scale), and sex (males in blue, females in red) of the 35 bovid populations. The size of the points corresponds to their weight in the analysis, except *Madoqua kirkii* (in gray) and *Redunca fulvorufu*la (in black) for which the weight is very low

The PC1 explained ca. 74% of the total variation in ontogenetic allometric intercept, slope, and proportional threshold. Negative scores on the PC1 characterized ontogenetic allometry with steep slope (high *β*_1_) and low values for the intercept and the threshold (Figure S3). The analysis of the effects of body mass, sex, and the range of body mass covered by the data on PC1 scores revealed a systematic effect of these three variables on patterns of ontogenetic allometry (Table 2). With an increasing body mass, ontogenetic allometries become shallower (lower slope) with higher intercept and later threshold (Figure 3). The statistically significant interaction between sex and species body mass suggests a stronger effect of body mass on ontogenetic allometry in females, even after removing the two outliers *M. kirkii* and *R. fulvorufula* (Table 2, Figure 3). A pairwise *t* test on the nine populations for which data from both sexes were available reveals no statistically significant differences in ontogenetic allometry between sexes (PC1 score females minus males = −0.090; 95% CI: −0.956; 0.776; *t* = −0.24; *df* = 8; *p*‐value = .82) females having a lower PC1 scores than males in four out of nine species. Finally, the range of body mass covered by the data had a positive effect on the ontogenetic allometric slope. However, there was no correlation between the species body mass and the range of body mass covered by the data (Pearson's correlation coefficient = 0.068; 95% CI: −0.271; 0.393; *t* = 0.39; *df* = 33; *p*‐value = .70).

**Table 2:** Parameter estimates from the linear model of the relationship between the first principal component of the PCA (including the allometric intercept *α*, the allometric slope *β*_1_, and the proportional threshold Tp) and adult body mass, sex, and body mass range with species as random effect

Variables	Fixed effects			Random effects		
	*β*	95% CI	*t*‐value		*N*	Var.
(A)						
Intercept	−0.401	−2.448; 1.646	−0.38	Species	19	0.493
Adult body mass	**0.644**	0.234; 1.055	3.08	Residuals		27.013
Sex (males)	**1.435**	0.109; 2.762	2.12			
Body mass range	**−0.021**	−0.038; −0.004	−2.37			
Adult body mass: Sex (males)	**−0.409**	−0.691; −0.126	−2.83			
(B)						
Intercept	−0.817	−3.828; 2.193	−0.53	Species	17	1.097
Adult body mass	**0.848**	0.246; 1.450	2.76	Residuals		55.066
Sex (males)	1.627	−0.274; 3.527	1.68			
Body mass range	**−0.028**	−0.053; −0.002	−2.13			
Adult body mass: Sex (males)	**−0.483**	−0.887; −0.078	−2.34			

Note

For random effect, the variance (Var.) is given. The analysis performed with (A, N = 35 populations) and without (B, N = 33 populations) the two extreme data corresponding to males of *M. kirkii* and *R. fulvorufula*, provided similar results. Parameters statistically different from 0 are reported in bold.

## 4 DISCUSSION

Our findings show that ontogenetic allometry between horn length and body mass varies across bovid species and is influenced by the adult body size of the species. In small species, horns grow at a much faster rate than body mass, but stop growing early compared to body mass. In large species, the ontogenetic allometry remains steeper than isometry, but horns grow much slower relative to body mass than in the small species, and this growth carries on for a longer period, sometimes as long as body mass increases. These differences in ontogenetic allometry generate a nonlinear evolutionary allometry similar to the one previously reported (Tidière et al., 2017). It is noteworthy that the evolutionary allometries in both sexes obtained with the current dataset are very similar to those reported by Tidière et al. (2017)'s analysis performed on 91 and 54 species for males and females, respectively (Table S3 and Figure 2).

Despite the emblematic status of bovids horn in ecological and evolutionary studies (Andersson, 1994; Darwin, 1871; Emlen, 2008; Geist, 1966; Lincoln, 1994), we were able to find data on growth or ontogenetic allometry of horn length for only 19 out of the 137 extend bovid species. Considering that females have horns in about 70% of these species (Lundrigan, 1996), this means that we were able to find data for only 30 (i.e., 14%) out of the 233 possible cases, with a skew toward small‐ and medium‐sized species. Furthermore, although data on body mass covered a large proportion of the range from birth to adult age (median coverage of 79%), limited coverage due to missing data for young age classes generated shallower slopes due either to the effect of measurement/biological error or to the decreasing growth rate of horns later in life. However, the range of body mass covered by each study was not correlated with the adult body mass of the species, and we are confident that our results on the changes in ontogenetic allometry with body mass are not an artifact of these sampling limitations.

Many factors affecting the evolution of horn size and shape in male and female bovids have been identified (Bro‐Jørgensen, 2007; Caro, Graham, Stoner, & Flores, 2003; Geist, 1966; Lundrigan, 1996; Packer, 1983; Stankowich & Caro, 2009). In several instances, these factors covary with body size. For example, ecological factors that influence both horn size and shape, such as habitat openness (Stankowich & Caro, 2009) or competition for food resources (Roberts, 1996), also tend to covary with species size. Small species generally inhabit closed habitat (e.g., forest), while large species generally use open habitat (e.g., savannah or prairies) (Jarman, 1974). Moreover, male fighting behaviors that also influence horn size and shape are expected to covary with species body size (Lundrigan, 1996) and could be a potential driver of the nonlinear pattern of evolutionary allometry highlighted by our results. In small species, such as common duiker, stabbing fight is associated with small spike‐like horns. In medium‐sized species, ramming behavioral fight is associated with robust and often recurved horns (e.g., mouflon, Alpine ibex). In larger species, such as the greater kudu (*Tragelaphus strepsiceros*), fighting behavior such as fencing or wrestling is generally associated with relatively thinner and straighter horns. Finally, mating system and the intensity of sexual selection that both influence the relative horn size in male bovids also covary with body size because sexual selection is more intense in large species that are predominantly polygynous or promiscuous such as bison than in small monogamous species such as duikers (Jarman, 1983; Pérez‐Barbería, Gordon, & Pagel, 2002; Tidière et al., 2017).

In small species that often display a mating tactic based on territoriality (Jarman, 1974), horns are expected to protect individuals against predators and to be used to defend the territory early in life when first reproduction occurs (Geist, 1966; Jarman, 1974). In these species, horns grow very rapidly for a short period. For medium‐sized species, including mountain ungulates, sexual selection is generally strong and involves intense fights among males that become active in the rut after a relatively long period of growth. In these species, horns may be used simultaneously as weapon, shock absorber during physical confrontation between males, or display organ (Geist, 1966). Males with the largest horns are generally dominant and have higher reproductive success (Bergeron, Grignolio, Apollonio, Shipley, & Festa‐Bianchet, 2010; Preston, Stevenson, Pemberton, Coltman, & Wilson, 2003). These different functions require the production of simultaneously large and robust horns able to resist the extreme forces resulting from fight (Alvarez, 1990; Kitchener, 1988). In these medium‐sized species, steep ontogenetic allometries are prolonged as long as body mass increases, resulting in particularly large horns relative to body mass (Tidière et al., 2017). Among the largest species, body mass and age are the main factors affecting male mating success (Wilson, Olson, & Strobeck, 2002; Wolff, 1998). Although horn size in these species may be used by females to assess the age of courting males, sexual selection directly targets body mass and not horn size. This hypothesis is indirectly supported by the lack of among‐species relationship between horn length and body mass and by the limited sexual size dimorphism in horn length observed in very large species (Tidière et al., 2017). Ontogenetic allometries in these species (>250 kg) are characterized by a shallow allometric slope and a late threshold value, which corresponds to an increase in horn size sometimes sustained as long as the increase in body mass. This growth tactic would make horn length a reliable indicator of age in the largest species, even at old ages. Thus, although mostly circumstantial, these observations suggest that the nonlinear evolutionary allometry between male horn length and body mass among bovids results from adaptive evolution of ontogenetic allometry in response to size‐specific selection on horn and body mass.

Alternatively, the shift from steep to shallow ontogenetic allometry with increasing species mass may prevent large species to develop horns with maladaptive proportions (Gould, 1966). Because large species usually grow for long period of time, steep ontogenetic allometry would produce particularly large horns that may be too costly to carry (Vander Linden & Dumont, 2019). Although Gould (1966) suggested this hypothesis for both ontogenetic and static allometry, it has never been tested for ontogenetic allometry, and evidence for a change in static allometry with body size is inconclusive (Emlen & Nijhout, 2000; Gould, 1966; Knell, Pomfret, & Tomkins, 2004; McCullough et al., 2015; Voje et al., 2014). Yet, as an illustration of this hypothesis, we calculated the size of the horns that *S. caffer* males (max body mass = 718 kg) would carry if they had an ontogenetic allometry similar to *C. ibex* males (max body mass = 109 kg). Using the estimated parameters obtained for *C. ibex* males (see Table 1), males *S. caffer* produce horns of ca. 95 cm when adult. With an ontogenetic allometry similar to *C. ibex*, those horns would reach ca. 12 m long (we used here the allometric slope and the intercept for *C. ibex* with the maximum body size of *S. caffer*). Although naïve, this calculation supports the idea that steep ontogenetic allometry would produce horns with extreme, most likely maladaptive, size in the largest species. If this was the reason for the shift in ontogenetic allometry, this study would be the first providing support to Gould's claim concerning the correlation between size and ontogenetic allometric slope.

Mass‐specific horn length in bovids seems to result from a combination of natural (in both sexes) and sexual (in males) selection that changes with species body mass and affects patterns of ontogenetic allometry. Although the quality of our data prevented us from estimating the relative contribution of the different parameters (intercept, slope, and threshold) to the changes in ontogenetic allometry, our results supports the idea that ontogenetic slopes evolve across species (Figure 1c). This does not necessarily imply that ontogenetic allometric slopes are highly evolvable, however. Indeed, if females tended to have shallower ontogenetic allometry with later threshold than males within species, these differences remained limited despite sometime strong sexual dimorphism in horn size. Although this result suggests a possible evolutionary constraint generated by intrasexual genetic correlation (Darwin, 1871; Lande, 1980), it remains speculative due to the limited amount of data available. Further studies on the genetic variation in ontogenetic allometry are necessary to better understand the evolution of static and evolutionary allometries.

## CONFLICT OF INTEREST

None declared.

## AUTHORS CONTRIBUTION

**Morgane Tidière:** Conceptualization (lead); data curation (lead); formal analysis (lead); investigation (lead); methodology (lead); writing – original draft (lead); writing – review & editing (lead). **Jean‐Michel Gaillard:** Conceptualization (equal); data curation (equal); investigation (equal); methodology (equal); writing – original draft (equal); writing – review & editing (equal). **Mathieu Garel:** Data curation (equal); methodology (equal); writing – review & editing (equal). **Jean‐François Lemaitre:** Conceptualization (equal); data curation (equal); investigation (equal); writing – review & editing (equal). **Carole Toïgo:** Data curation (equal); methodology (equal); writing – review & editing (equal). **Christophe Pélabon:** Conceptualization (lead); data curation (lead); formal analysis (lead); funding acquisition (lead); investigation (lead); methodology (lead); writing – original draft (lead); writing – review & editing (lead).

## Supporting information

Appendix S1‐S2

## Data Availability

All data used in this study can be found in DRYAD https://doi.org/10.5061/dryad.qz612jmb4 (R script corresponding is available in Appendix S2), as well as in Table 1.
