# Supplementary material for: Variation in the ontogenetic allometry of horn length in bovids along a body mass continuum
Source: Ecol Evol. 2020 Mar 27;10(9):4104–14. doi: 10.1002/ece3.6181 (PMC7244813; doi:10.1002/ece3.6181)

SUPPLEMENTARY INFORMATION

**Variation in the ontogenetic allometry of horn length in bovids along a body mass continuum**

Morgane Tidière, Jean-Michel Gaillard, Mathieu Garel, Jean-François Lemaître, Carole Toïgo, and Christophe Pélabon

**RUNNING TITLE:** Ontogenetic allometry in Bovids

**KEY WORDS:** comparative analysis, development, ornament, sexual selection, ungulates, weapons

**Appendix S1. Illustration of the use of segmented regression vs. quadratic linear regression to compare the ontogenetic allometry between horn length and body mass between *Capra ibex* males and *Gazella subgutturosa* males.**

It is difficult to compare non-linear ontogenetic allometries when species sizes strongly differ. We illustrate this issue by comparing the ontogenetic allometry between *Gazella subgutturosa* and *Capra ibex* (Fig S1). Using quadratic linear regression, one can compare the allometric slope of the two species using the derivative of the quadratic models for a given body mass. For example, for a body mass of 16 kg, the allometric slope for *G. subgutturosa* is 1.89 compared with 1.23 for *C. ibex.* Such comparison is possible if species have relatively similar body mass but remains meaningless for species with mostly non-overlapping body mass as it is the case here. Alternatively, one can use segmented regression (red lines). In this case, we assume that the change in allometric slope is abrupt during ontogeny (the quadratic model assumes a constant change in allometric slope during ontogeny). The model selection using AIC between the quadratic model and the segmented regression shows in both species, the segmented regression describes better the data than a linear or quadratic regression.

**Figure Appendix S1. Ontogenetic allometry between horn length and body mass for *Capra ibex* males (grey) and *Gazella subgutturosa* males (black) on a log-log scale.** The blue lines represent linear (*C. ibex*) and quadratic (*G. subgutturosa*) models (see Table S1 for parameter estimates).

**Table Appendix S1. Parameter estimates for the linear/quadratic models and for the segmented regression models for males of two species.** See Figure Appendix S1 for the representation of the models.

| *Capra ibex* | *Gazella subgutturosa* |
| --- | --- |
| Linear / quadratic model | Quadratic model |
| Log(HL) = -1.08 + 1.23 log(BM)* | Log(HL) = -10.59 + 7.96 log(BM) – 1.09 (log(BM))^2^ |
| AIC = -84.96 | AIC = 94.07 |
|  |  |
| Segmented model |  |
| α = -1.18 ±0.13 | α = -5.07 ±0.25 |
| β_1_ = 1.26 ±0.03 | β_1_ = 2.98 ±0.10 |
| β_2_ =0.28 ±0.54 | β_2_ =0.50 ±0.23 |
| TBM = 4.46 (4.38; 4.69) | TBM = 2.81 (2.78; 2.87) |
| AIC = -86.10 | AIC = 64.90 |

*Quadratic term not statistically significant (P = 0.67). We present here the linear regression.

**Appendix S2. Code used to estimate the threshold and its 95% confidence interval by maximum likelihood (Ulm and Cox 198****9) in R. Dataset corresponding is available in** <https://doi.org/10.5061/dryad.qz612jmb4>

dataset = read.table('dataset_Tidiereetal2020.txt',header=T,dec=".")

#######################################################

######## —— Species, sex and population definition —— ########

sp <- 'Bison_bonasus'

sx <- 'male'

pop <- 'Bialowezia'

data2 <- subset(dataset, dataset$species==sp & dataset$sex==sx & dataset$population==pop &

dataset$horn_length_cm!=0 & dataset$body_mass_kg!='NA' & dataset$horn_length_cm!='NA')

########################################################################

############ ------- Definition of the best threshold (Olivier Gimenez) ----- ############

BM<-with(data2, log(body_mass_kg))

#Function to define the threshold

threshold1<-function(T1, dataset) {

attach(data2)

BM.1 <- BM.2 <- BM

BM.1[BM.1 > T1] <- T1

BM.2[BM.2 <= T1] <- 0

BM.2[BM.2 > T1] <- BM.2[BM.2 > T1] - T1

detach()

list(BM1.1 = BM.1, BM1.2 = BM.2)

}

#Definition of the range of possible thresholds

th <- seq(min(BM)+0.001, max(BM)-0.001, 0.01)

le <- length(th)

L <- vector()

#Definition of the maximum likelihood of model for each threshold defined in th

for(j in 1:length(th)) {

i <- th[j]

h <- BM

h[h>=i] <- i

lm0 <- lm(log(horn_length_cm) ~ h, data=data2)

L[j] <- logLik(lm0)

}

#Definition of the threshold corresponding to the model with the maximum likelihood (Ulm & Cox 1989)

graal = th[(1:length(th))[(max(L)==L)]]

T1<-round(graal,digits=3) # value of the threshold

### Definition of 95 confident interval values around the threshold ###

LLbt <- max(L) #log-likelihood of the best threshold

LLdelta <- LLbt - L #delta between best threshold and other thresholds

LLdelta2 <- 2*LLdelta

tabLL <- as.data.frame(cbind(LLdelta2, exp(th)))

tabLL2 <- subset(tabLL, tabLL$LLdelta2<2.71)

T1_95CI_low <- round(log(tabLL2[1,2]), digits=3) #95% CI lower value

T1_95CI_hig <- round(log(tabLL2[length(tabLL2[,1]),2]), digits=3) #95% CI higher value

### Table 1 values ###

round(exp(T1), digits=2)

round(exp(T1_95CI_low), digits=2)

round(exp(T1_95CI_hig), digits=2)

round((T1/max(BM)), digits=2)

exp(max(BM))

####################################################################

### —— Allometry definition fitting a segmented regression with two slopes —— ###

data2$BM1.1=threshold1(T1,data2)$BM1.1

data2$BM1.2=threshold1(T1,data2)$BM1.2

mod2S <- lm(log(horn_length_cm)~BM1.1+BM1.2, data2)

summary(mod2S)

**Table S1. Methods used to measure horn length according to the original study for the 19 Bovid species included in this study.** Horn length is generally measured from the tip to the base of the horn using a flexible ruler placed along the external curvature of the horn.

| **Reference** | **Species** | **Method description** |
| --- | --- | --- |
| Anderson 1982 | *Aepyceros melampus* | Follows the method described by Sachs (1967): Horns are measured from the base to the tip following the front curvature, and around the base of the horn. |
| Bassano and al. 2003 | *Rupicapra rupicapra* | Follow the method described in Knaus and Schröder 1983: Horn length was measured along the frontal curvature. |
| Child and al. 1972 | *Damaliscus l. lunatus* | Measured from the length around the curve (see fig. 9) |
| Cote and al. 1998 | *Oreamnos americanus* | Total length of the horn measured with a measuring tape along the outside curve. |
| Fandos and Vigal 1988 | *Capra pyrenaica* | Horn length measured using a string applied to the anterior crest of the sheath and then measuring the string. |
| Geist and Walther 1974 | *Syncerus caffer* | Not found |
| Hutchison 1970 | *Madoca kirkii* | Length of horn along frontal surface. |
| Krasińska and Krasiński 2002 | *Bison bonasus* | Measured with a tape along the external curve of the horn from basis to end (see fig. 1). |
| Locati and Lovari 1991 | *Rupicapra rupicapra* | No description |
| Norton and Fairall 1991 | *Redunca fulvorufula* | Not found |
| Parkes and Tustin 1989 | *Hemitragus jemlahicus* | The length of one horn was measured from its tip along the top edge to the point closest to the skull. |
| Prinsloo and Jackson 2017 | *Tragelaphus strepsiceros* | Not found |
| Robinette Archer 1971 | *Gazella thomsonii* | The horns were measured along the frontal curvature. |
| Robinson et al 2006 | *Ovis aries* | Horn size was measured as the length of the horn (in mm) from the base along the outer curvature of the spiral to the tip. |
| Wilson et al 1984 | *Sylvicapra grimmia* | Horn length was measured along the front surface of both horns. |
| Wronski et al 2010 | *Gazella gazella*  *Gazella subguturosa*  *Gazelle dorcas* | Measured from horn base to horn tip following the lyrate shape of the horn. |

**References used in Table S1**

Knaus, W. and W. Schröder, 1983. – Das Gamswild. Paul Parey Verlag, Hamburg.

Sachs, R. 1967. Liveweights and body measurements of Serengeti game animals. African Journal of Ecology 5:24–36.

**Table S2. Pearson's correlations between the parameters of the segmented regression models estimating the ontogenetic allometry in males and females of 19 bovid species** (α: the allometric intercept, β_1_: the allometric slope before the threshold, T_P_: the threshold expressed as proportion of the maximum body mass). The upper part presents the correlation with all the data while the lower part presents the data without *Madoqua kirkii* and *Redunca fulvorufula*.

|  |  | **α** | **β_1_** |
| --- | --- | --- | --- |
| **All sexes, populations and species**  N = 35 populations | **β_1_** | -0.80  p-value < 0.001 |  |
|  | **Tp** | 0.42  p-value = 0.012 | -0.58  p-value < 0.001 |
| **Without *M. kirkii* and *R. fulvorufula***  N = 33 populations | **β_1_** | -0.92  p-value < 0.001 |  |
|  | **Tp** | 0.50  p-value = 0.003 | -0.56  p-value < 0.001 |

**Table S3. Parameter estimates from evolutionary models (on a log-log scale) in males and in females of Bovid species obtained in this study and reported by Tidière et al. (2017).** See also Figure 2.

|  | **Study** | **Variables** | **β** | **95%CI** | **t-value** | **Adjusted-R^2^** |
| --- | --- | --- | --- | --- | --- | --- |
| **Males** | **Our study** | Intercept | -0.165 | -1.894;1.564 | -0.19 | 0.60 |
|  | n=21 populations, 19 species | Body mass | 1.458 | 0.623;2.293 | 3.42 |  |
|  |  | Body mass^2^ | -0.118 | -0.216;-0.020 | -2.37 |  |
|  | **Tidière et al. 2017** | Intercept | 0.296 | -0.526;1.116 | 0.70 | 0.53 |
|  | n=91 species | Body mass | 1.023 | 0.672;1.374 | 5.71 |  |
|  |  | Body mass^2^ | -0.058 | -0.098;-0.018 | -2.86 |  |
| **Females** | **Our study** | Intercept | 1.957 | 1.312;2.603 | 5.94 | 0.48 |
|  | n=14 populations, 11 species | Body mass | 0.315 | 0.144;0.487 | 3.61 |  |
|  | **Tidière et al. 2017** | Intercept | 0.316 | -0.561;1.194 | 0.71 | 0.48 |
|  | n=54 species | Body mass | 0.661 | 0.478;0.844 | 7.06 |  |

**Figure S1. Segmented regression model was fitted on raw data for males (blue) and females (red) to define ontogenetic allometry of horn size in the 19 bovids species used in the study.**

**Figure S2. Distribution of the ontogenetic allometric intercept α (A), slope β_1_ (B) and threshold Tp (C) for males (blue) and females (red) of the 19 bovid species (35 populations).** Extreme values are obtained for *M. kirkii* males (grey triangle) and *R. fulvorufula* males (black triangle). Analyses have been made with and without these two species to ensure the reliability of the results.


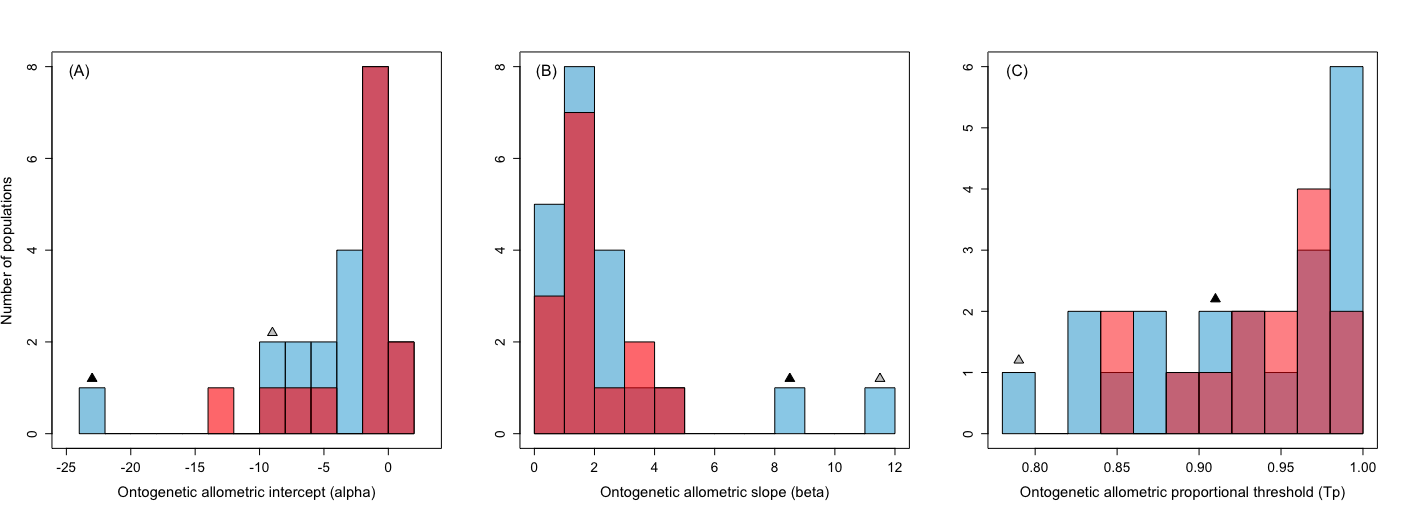


**Figure S3. Representation of the normed Principal Component Analysis including ontogenetic allometric intercept alpha, slope beta and threshold Tp for males (in blue) and females (in red) of the 19 (A) and 17 (B) bovid species included in the study.** Allometric slope is negatively correlated to the intercept and the threshold: low value of the first axis indicates a steep and short horn growth relatively to body mass while high value represents a shallow and late stopped horn growth.


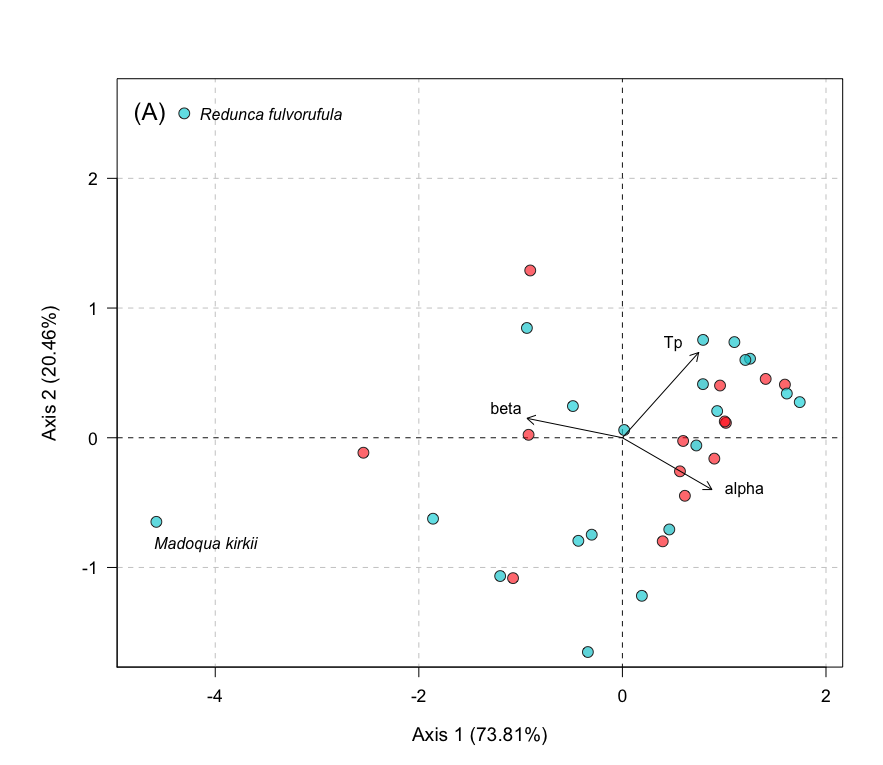

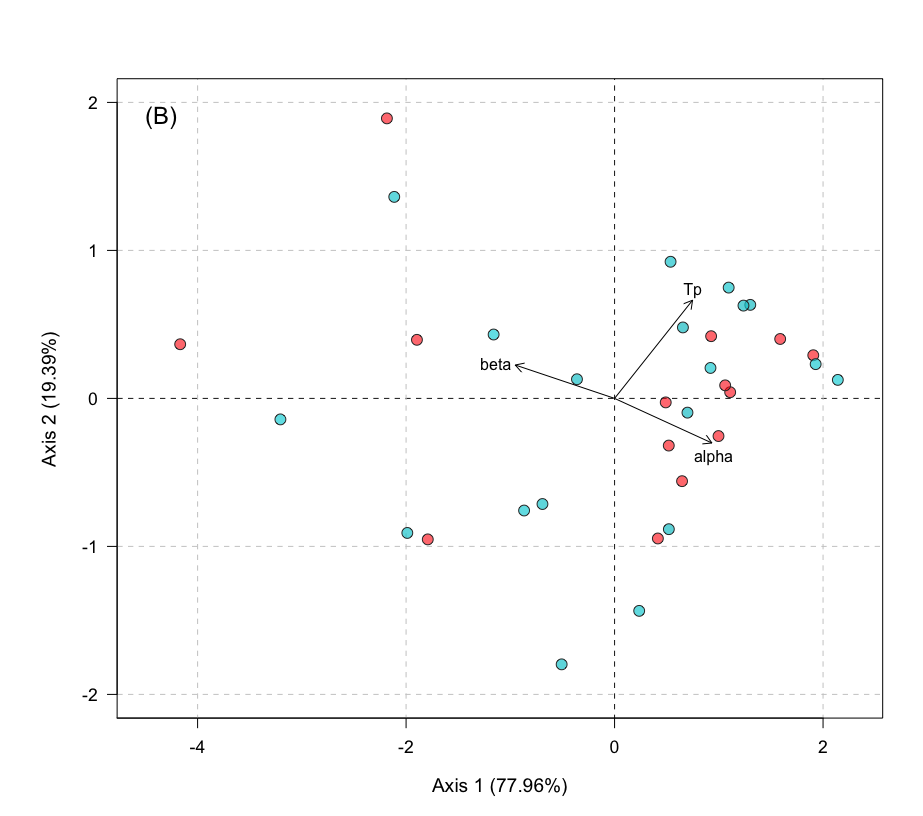

Supplement: Supplementary file 1 — Appendix S1‐S2 [file ECE3-10-4104-s001.docx]
